# Influence of Individual Ions on Silica Nanoparticles Interaction with Berea Sandstone Minerals

**DOI:** 10.3390/nano9091267

**Published:** 2019-09-05

**Authors:** Aly A. Hamouda, Rockey Abhishek

**Affiliations:** Institute of Energy and Petroleum Engineering, University of Stavanger, 4036 Stavanger, Norway

**Keywords:** silica, nanoparticles, adsorption, fluid/rock interaction, Mg^2+^, Na^+^, SO_4_^2−^

## Abstract

Nanofluids are prepared by dispersing silica nanoparticles in aqueous media (brines). The purpose of this work is to address brine/rock interactions in presence of nanoparticles. Our previous studies have shown that silica nanofluids are effective in reducing formation damage in sandstone reservoirs. This study addresses effect of individual ions on dispersed silica nanoparticles’ interaction with Berea Sandstone minerals. The selected ions are Mg^2+^, SO_4_^2−^ and Na^+^, in MgCl_2_, Na_2_SO_4_ and NaCl, which are the major constituents of seawater. Three flooding stages for Berea Sandstone cores were followed. The first flooding stage was without nanoparticles, the second one was a slug of the nanoparticles with tracer and the third stage was a post-flushing of the core with the respective ion. The effluent tracer concentration, nanoparticle content, ion concentrations and pH reflect the effect of individual ions on nanoparticle/mineral interaction which were used for suggesting possible interaction mechanisms. Presence of Mg^2+^ and SO_4_^2−^ ions improved the adsorption of nanoparticles on minerals, however the effect of Na^+^ was lesser. In general, in all the cases nanoparticles reduced the mineral dissolution and associated fine migration/possible formation damage.

## 1. Introduction

The fall in oil prices over the last few years has led to increased focus on developing efficient enhanced oil recovery (EOR) techniques that can make oil production from maturing oil fields economically attractive [1,2,3]. Silica nanoparticles (NPs) dispersed in various fluids have emerged as an attractive option since they have the potential to effective at very low volume concentrations [4] thereby reducing the cost of applying this technology. Potential applications of silica nanofluids (NFs) include: (a) surface modification to alter wettability [5,6,7]; (b) Interfacial Tension(IFT)reduction [8,9]; (c) increasing structural disjoining pressure for increasing oil recovery [10,11,12]; (d) mitigating formation damage [13,14,15,16]; and (e) improving the performance of low salinity water floods [17,18,19].

Water flooding is a well-established secondary oil recovery method for reservoir pressure maintenance post-primary production phase. Most offshore oil reservoirs are flooded with seawater due to easy availability and low cost. It is well established in the literature that ionic composition of injection brine affects the crude oil brine system [20,21,22]. Lowering injection brine salinity is a popular EOR method [23,24], but it may have some detrimental effects in the reservoir. Khilar and Fogler [25] proposed a limit on brine salinity for injection in sandstones known as critical salt concentration (CSC). They proposed that if the injected salinity below was below CSC, there may be a release of clay particles and this may cause formation damage. Formation damage by lowering injection brine salinity has also been reported by other research groups [26,27,28]. 

Silica nanofluids are prepared by dispersing silica NPs in appropriated brines. Therefore, is stands to reason that the ionic composition of brine used to prepare the nanofluid would itself affect fluid/rock interaction in the reservoir. Previous work in our lab identified that adding silica NPs to low salinity water can reduce fine migration and mineral dissolution in Berea Sandstone [18]. Low salinity water is in itself a promising EOR method. However implementing this method can incur high investment involved in setting up desalination plants and transport infrastructure especially in off shore environments [29]. Therefore, adding silica NPs to cheaply available seawater presents a more cost-effective solution. Many researchers have reported that silica NPs can be effective in increasing oil recovery from sandstones reservoirs dispersed in seawater [12,30,31,32,33]. Wettability alteration is the major mechanism attributed to the effectiveness of silica nanofluid [5,6,7]. Wettability alteration depends on the surface modification of Berea Sandstone due to the adsorption of silica NPs on mineral surface. Therefore, the adsorption behavior critically controls the effectiveness of silica nanofluid as an EOR. Monfared, et al. [34] showed that for single salt NaCl brine NP adsorption on calcite mineral increases with salinity. Previous work in our lab [35] investigated the kinetic adsorption of silica NPs on Berea Sandstone as the salinity of multicomponent brines was increased from DIW (no added salts) to low salinity (1:10 diluted seawater) and finally high salinity (seawater). We reported that both rate and equilibrium adsorption increased with salinity. Seawater is a multicomponent brine and therefore the different ions present in seawater have a combined and complicated effect on the performance of the nanofluid flood. Hence, it is important to address the role played by the major ion present in seawater, as this would enable operator to design a more effective brine for dispersing silica NPs and preparing more effective nanofluid for EOR application. However, to the best of our knowledge, the effect of silica NPs dispersed in seawater on fluid/rock interaction and its adsorption in Berea Sandstone has not been addressed in the literature.

The objectives of this work are to address individual ions such as magnesium, sulphate and sodium on NP adsorption and NP/mineral interaction associated with the injection of silica nanofluids in Berea Sandstone cores. These ions were selected since they are the major constituents of seawater and are therefore expected to have direct effect on the performance of silica nanofluid injection. Single ion brines of these ions were considered as stabilizing fluids for NPs. This work contributes to isolate and understand the influence of the individual ions on the NPs/mineral interaction and their mechanism(s).

In the following sections, the materials used for this study and the experimental methodology for core flooding are outlined followed by the results. The results include the detailed quantitative analysis of the effluent characterization. The produced effluent characterization included determining NPs concentration, ion concentration and pH. Additionally, the particle size of the NPs dispersed in various brines and the zeta potential of the NPs and crushed Berea powder dispersed in different brines was measured. The data from these measurements were used to determine the electric double layer interaction between the silica NPs and the Berea. Finally, the summary and conclusions of this study are presented in the final section.

## 2. Materials and Methods

The Silica NPs (DP 9711) used in this work was provided by Nyacol Nano Technologies (Ashland, MA, USA) at 30 wt% concentration, dispersed in deionized water (DIW). The stock fluid was supplied at pH 3. The manufacturer claimed NP size was about 20 nm. The nanofluids used in this study were prepared from the stock fluid by diluting it with appropriate brines. Synthetic seawater (SSW) brine was prepared by dissolving NaCl (AnalaR NORMAPUR, VWR Chemicals, Oslo, Norway), Na_2_SO_4_ (Honeywell Fluka, Oslo, Norway), NaHCO_3_ (Honeywell Riedel-de-Haën, Oslo, Norway), KCl (Sigma-Aldrich, Oslo, Norway), MgCl_2_·6H_2_O (AnalaR NORMAPUR, VWR Chemicals, Oslo, Norway) and CaCl_2_·2H_2_O (AnalaR NORMAPUR, VWR Chemicals, Oslo, Norway) at appropriate concentration in DIW and filtering the brine before use with 0.22 μm filter paper. The single salt brines were prepared by dissolving NaCl, MgCl_2_·6H_2_O and Na_2_SO_4_ in DIW for Na Brine, Mg Brine and SO_4_ brine respectively. The single salt brines were also filtered with 0.22 μm filter paper. The ionic composition and ionic strength of the brines used in this study are listed in Table 1. Berea Sandstone outcrop cores obtained from Koucurek Industries Inc., Caldwell, TX, USA were used as the porous media for performing injection experiments. Zetasizer Nano ZSP from Malvern Instruments (Malvern, Worcestershire, UK) was used to measure the average particle size and zeta potential of the NPs and the zeta potential of the Berea mineral dispersed in different brines and Berea mineral which was modified by silica NPs. The detailed method for modifying the Berea mineral with silica NPs and measuring zeta potential can be found elsewhere [18].

### 2.1. Nanoparticle Slug Injection

**Figure 1:**
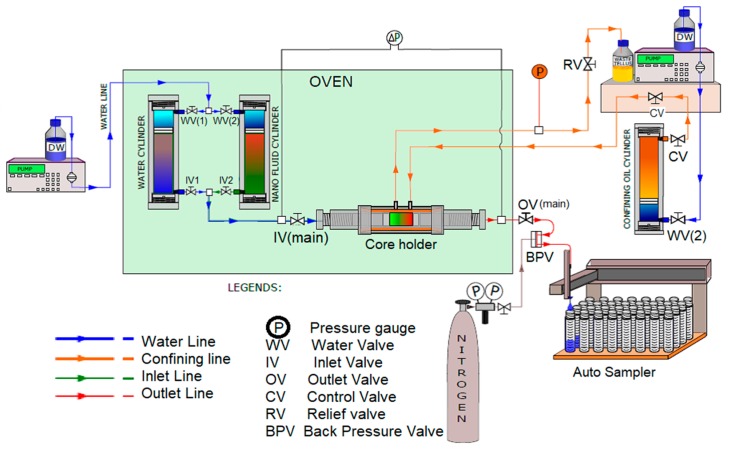
Schematic of the core flooding setup.

The objectives of the tests were to study the adsorption profile of the injected NPs and the interaction between NPs and the minerals present in Berea Sandstone. The schematic of the core flooding setup used this study is shown in Figure 1. Prior to loading the cores in the flooding setup, the cores were dried in a vacuum oven at 100 °C until the weight stabilized. Thereafter, the core was vacuum saturated with SSW. The difference between the dry weight and the saturated weight was used to determine the pore volume (PV) of the core. The SSW saturated core was then loaded into the core holder and a confining pressure of 25 bar was applied. After injecting several PVs of brine (pre-flush), 1.5 pore volume (PV) of slug with LiCl (AnalaR NORMAPUR, VWR Chemicals, Oslo, Norway) tracer was injected. Thereafter, the injection was switched to the original fluid to conduct a post-flush. Both brine and nanofluid slug injection was performed at a constant flow rate of 10 PV/day and at room temperature. Details of the experiment are listed in Table 2.

The effluents were analyzed for NP concentration, ion concentration and pH. NPs concentration measurement was performed using a dual beam UV-Vis 1700 spectrophotometer from Shimadzu Corporation (Nakagyo-ku, Kyoto, Japan). The concentration of the ions produced in the effluents from the flooding experiments was determined by using a Dionex ICS-5000 ion chromatograph from Thermo Fisher Scientific (Waltham, MA, USA).

### 2.2. Electric Double Layer Interaction

The theory of surface forces was used in this work to estimate the electric double layer interaction between the NPs and the Berea minerals. There is a significant size difference between the NPs and the mineral surfaces. Therefore, the curvature of the mineral surface can be neglected so that the double layer interaction between the NPs (sphere) and mineral (plate) can be modelled based on the sphere–plate collector geometry [36]. The electric double layer interaction (*V_EDL_*) can be calculated as [34,37]:

(1) $$V_{EDL}\left(h\right)=\pi \varepsilon_{0}\varepsilon_{1}\kappa (\zeta_{p}^{2}+\zeta_{s}^{2})\overset{a_{p}}{\underset{0}{\int }}(\begin{array}{c} -coth\left[\kappa \left(h+a_{p}-a_{p}\sqrt{1-{\left(r/a_{p}\right)}^{2}}\right)\right]\\ +coth\left[\kappa \left(h+a_{p}+a_{p}\sqrt{1-{\left(r/a_{p}\right)}^{2}}\right)\right]\\ +\frac{\zeta_{p}\zeta_{s}}{\zeta_{p}^{2}+\zeta_{s}^{2}}csch\left[\kappa \left(h+a_{p}-a_{p}\sqrt{1-{\left(r/a_{p}\right)}^{2}}\right)\right]\\ -\frac{\zeta_{p}\zeta_{s}}{\zeta_{p}^{2}+\zeta_{s}^{2}}csch\left[\kappa \left(h+a_{p}+a_{p}\sqrt{1-{\left(r/a_{p}\right)}^{2}}\right)\right] \end{array})r\cdot dr$$

where *ε*_0_ and *ε*_1_ represent the vacuum permittivity and relative permittivity of water; *h* is the separation distance between the surfaces; *ζ_p_* and *ζ_s_* are the surface potentials of the NP and mineral, respectively, which can be approximated as their respective zeta potential; *a_p_* is the NP radius; and *κ* is the inverse Debye length. For brines, the inverse Debye length depends on the salinity of the intervening medium:

(2) $$\kappa^{-1}=\sqrt{\frac{\varepsilon_{0}\varepsilon_{1}k_{B}T}{2e^{2}N_{a}I}}$$

where *e* is the elementary charge of an electron (C), *k_B_* is the Boltzmann constant, *N_a_* is the Avogadro constant, *T* is temperature and *I* is the ionic strength of the medium.

(3) $$I=\frac{1}{2}\sum c_{i}Z_{i}^{2},$$

where, *c_i_* is the ion concentration of the *i^th^* species and *Z_i_* is the valence number of the *i^th^* species as listed in Table 1. Finally, the non-dimensional double layer interaction energy (*V_EDL,ND_*) can be calculated as follows:

(4) $$V_{EDL,ND}\left(h\right)=\frac{V_{EDL}\left(h\right)}{k_{B}*T}.$$

## 3. Results and Discussions

Core flood investigations were conducted in this work to address the performance of silica NPs dispersed in single salt brines (MgCl_2_, Na_2_SO_4_ and NaCl) and SSW (used in some sections here as a reference). The effluent samples were analyzed for NPs concentration by UV-Vis. Ion concentrations and tracer (LiCl) presence in the effluent were determined by ion chromatogram and the pH level was measured. This section is divided into three subsections, NPs and tracer concentration profiles, double layer interaction and NPs/mineral interaction with possible mechanism(s). 

### 3.1. Nanoparticle and Tracer Profiles

**Figure 2:**
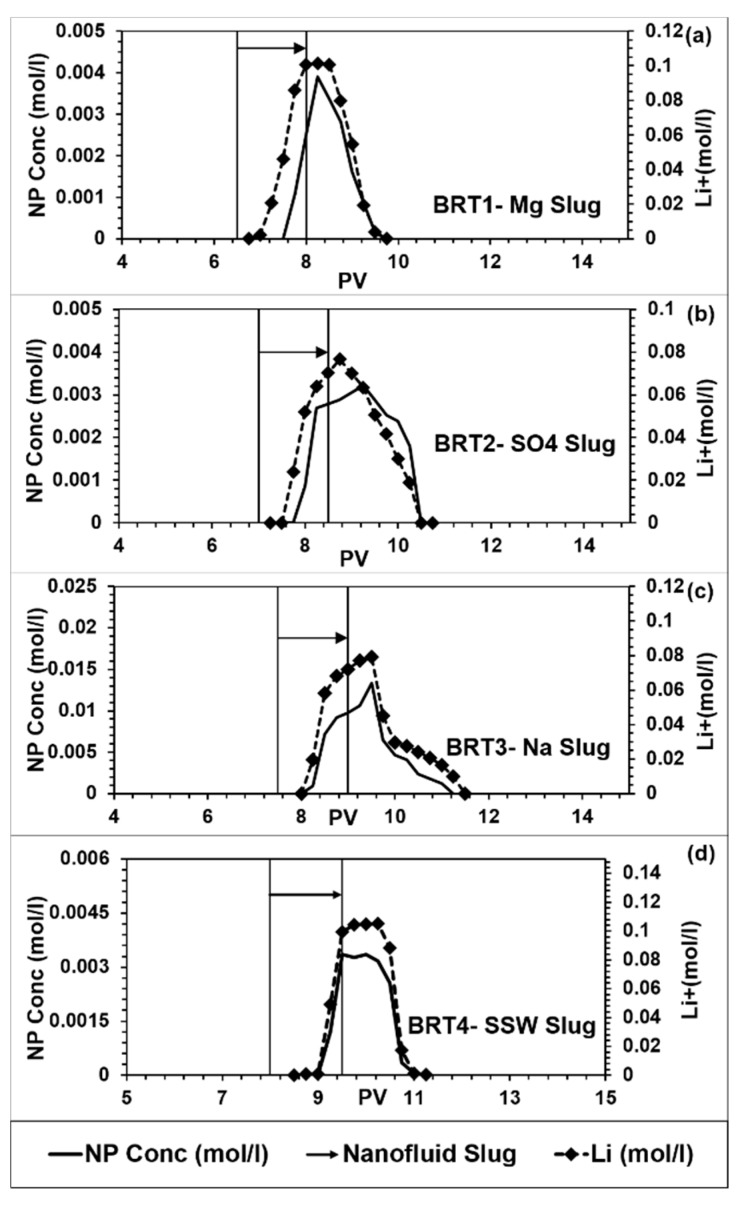
Nanoparticle (NP) and tracer concentrations from flooding of (**a**) BRT1; (**b**) BRT2; (**c**) BRT3; (**d**) BRT4.

The NPs and tracer profiles for the flood conducted in this work are shown in Figure 2. In our previous work [18] with dispersed NPs in low salinity water injected into Berea Sandstone, we observed significant NP production after tracer had ceased to produce. Figure 2 shows that percentage of retained/adsorbed NPs varied with the dispersing fluid. The amount of retained NPs in the core is estimated by integrating the area under the NPs production curves and comparing with the known injected amount of NPs into the core. The estimated amounts of NPs retained are shown in Table 3. 

**Figure 3:**
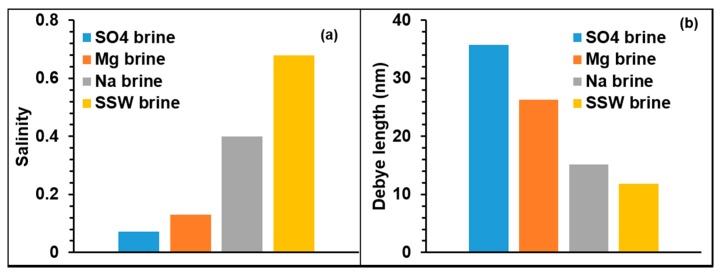
(**a**) Salinity for different brines; (**b**) Debye lengths for different brines.

**Figure 4:**
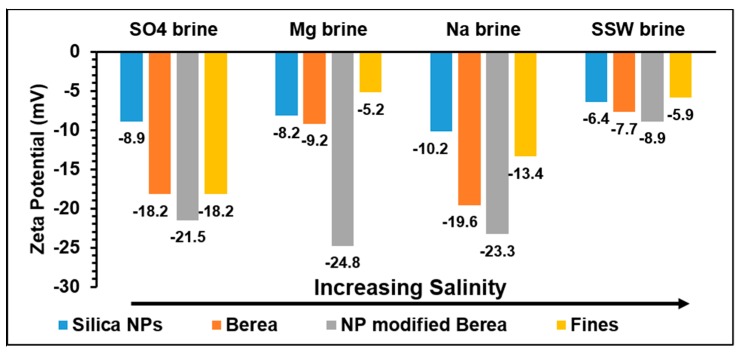
Zeta potential for the different fluids.

SSW and Mg brine flooding showed maximum NP retention/adsorption of about 81%. Flooding with SO_4_ brine showed an intermediate NP retention/adsorption of about 68% and the lowest NP retention/adsorption of about 34% was estimated for the case of Na brine flooding. Figure 3 shows the influences of the ionic strength (salinity) of the NPs dispersing brines on Debye length. Previous studies have shown that NP adsorption increases with salinity [34,37]. However, from Table 3, the level of NPs adsorption was similar (~80%) in the case of SSW and Mg as dispersing brines, in spite of the significant difference in their ionic strength. In addition, high salinity in the case of Na^+^ brine (salinity = 0.4) corresponds to the lowest adsorption (~34%) while SO_4_ brine with the lowest salinity (0.072) showed an intermediate NP adsorption (68%). Therefore, these results may indicate that the salinity of the NPs’ dispersing medium may not be the main factor that influences NP adsorption on mineral surfaces. The composition of the dispersing brines affects zeta potential of the interacting NP, fluid and mineral surfaces, as demonstrated in Figure 4. 

Figure 4 shows the zeta potential for NPs/brines, Berea/brines and modified Berea with NPs/brines. Zeta potentials for all brines used in this study were negative. The average zeta potential of NP-modified Berea for the single ion brines was about 23 ± 2 mV while SSW with multiple-ion composition exhibited the lowest zeta potential, and also had the highest amount of retained/adsorbed particles. From this point on in the manuscript, the term adsorption will be used. The term retained may give impression of core damage. The term adsorption is justified based on our previous work [16,17,18] where a decrease in pressure drop across the core was detected when flooding with silica NPs, rather than an increase in pressure drop which would be indicative of core damage. 

### 3.2. Double Layer Interaction

**Figure 5:**
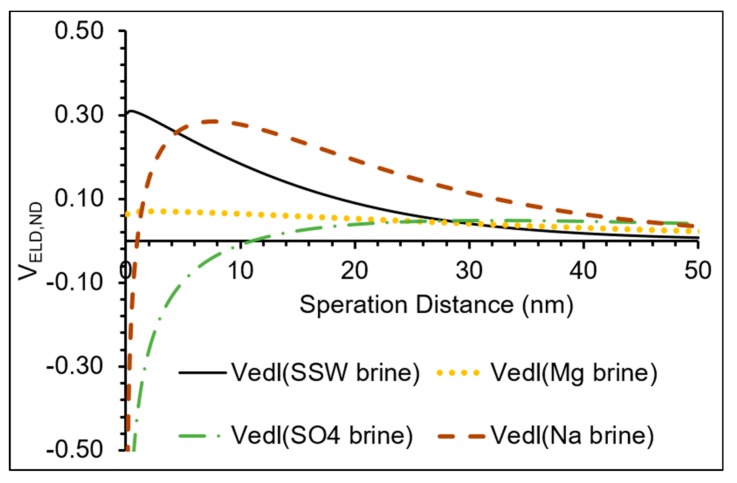
Double layer interactions for NPs/mineral surfaces in different brines.

Based on the Debye lengths (Figure 3b), NPs’ sizes dispersed in different brines (Table 4) and the zeta potential (Figure 4), NPs–mineral surfaces double layer interactions were estimated by Equation (1). The results are shown in Figure 5.

Figure 5 shows that the interaction is mostly repulsive in the case of Na brine, while it is less repulsive in the case of the other brines. It is interesting to note that in the case of Mg brine, the double layer interaction remains almost constant at all the separation distances. This is difficult to explain, however it may have been caused by a homogenous fluid around the mineral (approximately 10 nm from mineral surface) which caused the diffused product of the exchange reaction between Mg^2+^ and the calcium in the carbonate (cementing material in Berea) hence reducing the potential difference around the minerals. This may also explain the highest adsorption in the case of the Mg brine where NPs in Mg brine diffuse into the minerals, where the exchange between Mg^2+^/Ca^2+^ takes place, hence increasing the contact surface area between NPs and the silicate mineral surfaces. 

Delays of NPs’ breakthrough occurred at different time from the tracer’s breakthrough indicating possible interactions with the minerals (Figure 2). In the case of Mg brine, there was a delay of approximately 0.5 PV (Figure 2a) for NPs’ breakthrough to occur. A similar delay of NPs’ breakthrough of 0.5 PV also occurred in the case of SO_4_ brine (Figure 2b). The next subsection addresses possible mechanisms of interaction for individual brines. 

### 3.3. NPs/Mineral Interaction and Possible Mechanism(s)

**Figure 6:**
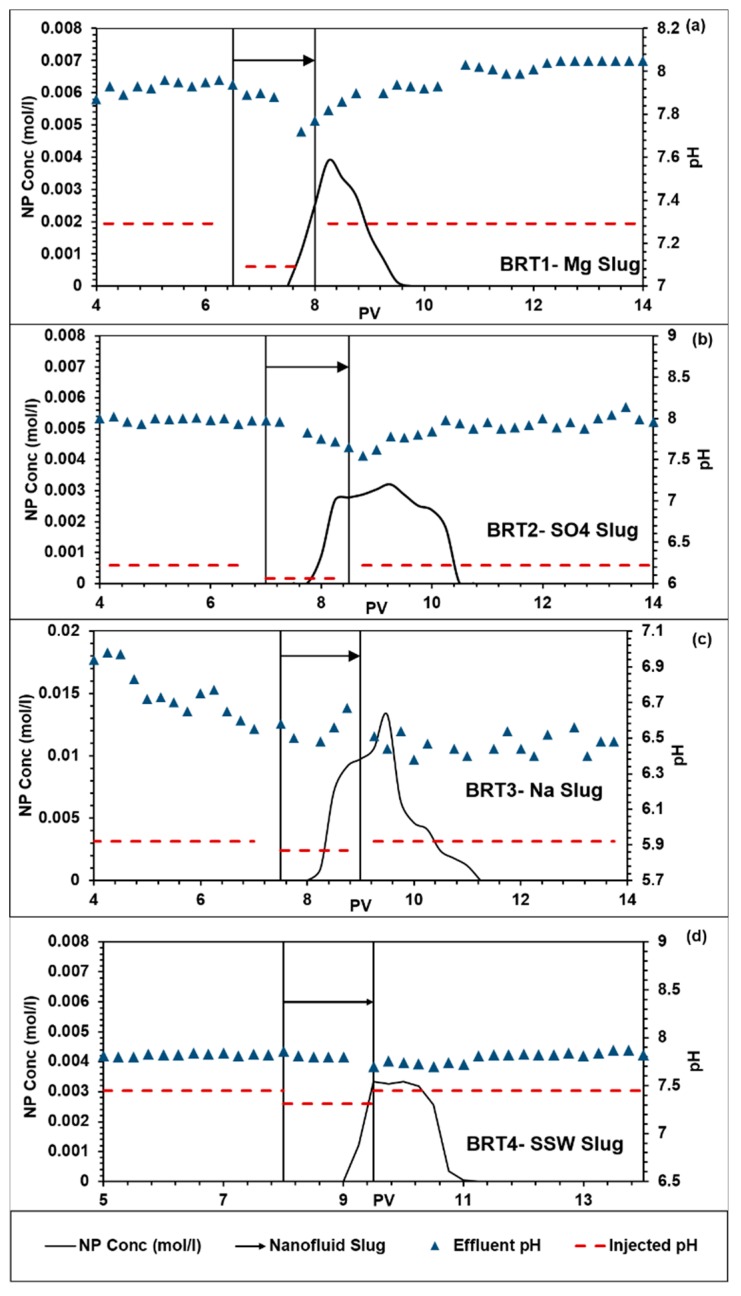
Effluent pH profiles for (**a**) BRT1; (**b**) BRT2; (**c**) BRT3; (**d**) BRT4.

**Figure 7:**
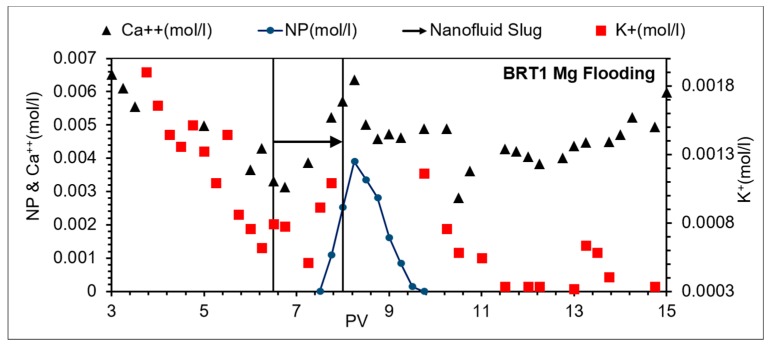
Effluent concentrations of NPs and ions for BRT1; Mg brine flooding.

**Figure 8:**
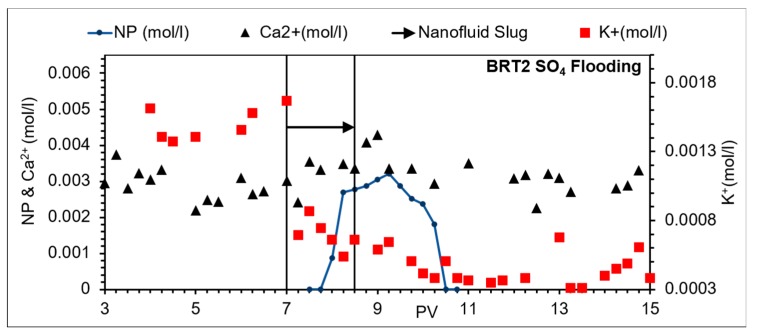
Effluent ion composition, BRT2; SO_4_ brine flooding.

**Figure 9:**
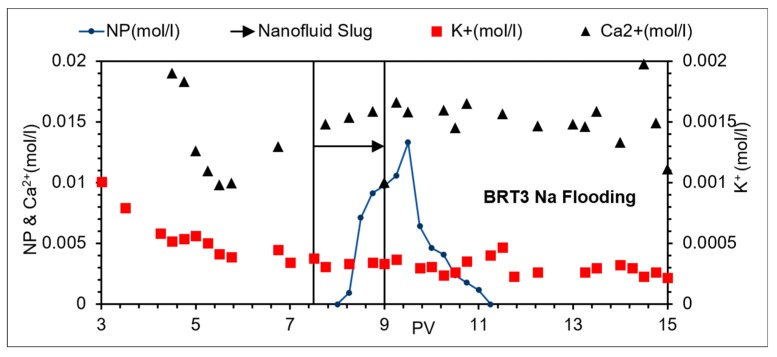
Effluent ion composition, BRT3 Na brine flooding.

This section addresses the interaction ion products and pH as a function of the stabilizing single ion brines to understand the mechanism(s) and the contribution of the different ions of the single ion brines in the interaction between NPs/mineral. It was decided, therefore, to exclude the multiple ion such as SSW, in this study. However, the multiple ion brines will be addressed in a different study. The effluent pH and ion concentration profiles for the floods are shown in Figure 6, Figure 7, Figure 8 and Figure 9. All flooding with nanoparticles showed large reductions of K^+^ ion concentrations. This perhaps indicates less mineral dissolution, hence possible reduction of fine migration or formation damage (discussed in detail later). 

Figure 7 (Mg flooding) shows a higher concentration of K^+^ in the effluent at the pre-flush stage of the flooding. There are two possible sources of K^+^ ions: (1) production of residual K^+^ from the initial core saturation fluid (SSW); and (2) production of K^+^ from K–feldspar dissolution. Previous studies [38] suggested the following equation for the K–feldspar dissolution. This equation also explains the rise of the pH from all brines (Figure 6) excluding (Figure 6c) which is for Na brine (addressed in a later subsection).

(5) $$\begin{array}{l} 4KAlSi_{3}O_{8}\left(s\right)\left(orthoclase\right)+22H_{2}0\left(aq\right)\\ \;\;\;\;\;\;\;\to Al_{4}Si_{4}O_{10}\left(s\right)\left(kaolinite\right)+8H_{4}SiO_{4}\left(aq\right)+4K^{+}\left(aq\right)+4OH^{-}\left(aq\right) \end{array}$$

#### 3.3.1. MgCl_2_ Brine

Using Mg brine as NPs’ stabilizing fluid showed a delay of NPs breakthrough of about 0.5 PV relative to the tracer breakthrough (as mentioned above); lowest zeta potential with Berea’s minerals; a constant double layer effect over a separation distance of about 40 nm; and pH before/after the NP slug being almost the same at approximately 8.0 (Figure 6a). 

The effluent Ca^2+^ (Figure 7) shows slightly higher average concentration (~0.005 mol/L) at the post-flush compared to about 0.004 mol/L prior to the slug, which may suggest an exchange reaction. The exchange reaction may lead to formation of dolomite or magnesian, depending on the Mg^2+^/Ca^2+^ ratio. Since, Mg^2+^ is constantly injected, it is difficult to relate the concentration ratio, therefore the exchange equation considered here is for the formation of dolomite, according to the following [39]:

(6) $$2CaCO_{3}\;\left(s\right)+Mg^{2+}\to CaMg{\left(CO_{3}\right)}_{2}+Ca^{2+}$$

The exchange reaction due to injection of Mg brine as a mechanism seems to explain the following observations: the highest adsorption and constant double layer for about 40 nm separation distance from the mineral surface (explanation is suggested in the previous subsection), and the higher concentration of calcium ions in the post-flush, which can be explained by Equation (6). 

#### 3.3.2. Na_2_SO_4_ Brine

Observations of flooding with Na_2_SO_4_ brine, as NPs stabilizing are as follows. A delayed NP breakthrough about 0.5 PV relative to the tracer breakthrough (indicating interaction between the NPs and mineral surfaces) was observed, and there was less repulsive double layer interaction than in the cases of other brines (Figure 5). The effluent had almost stable average pH at about 8 except within the slug window, where the pH value was reduced by about 0.5 (Figure 6b). The was an almost stable calcium concentration at about 0.003 mol/L (Figure 8). Zeta potentials for cation/minerals were more negative than that in the case of SO_4_^2−^ (Figure 4), opposite to what was expected.

Anions must be capable of accepting or donating protons to be adsorbed on minerals [40]. Sulphate adsorbs on kaolinite and releases OH^−^ [41]. Researchers suggested that the change of the surface charge (negative charge) is neutralized by participation of neighboring Al ions resulting in ring formation [42]. 

The suggested mechanism here is that SO_4_^2−^ ions adsorb on kaolinite and release OH^−^. These in turn increase the pH = 8 as observed in this work and keep it almost constant, since SO_4_^2−^ ions are injected. It partially reacts with Ca^2+^ ions. Ca^2+^ ions concentration remained almost same (0.003 mol/L) before and after the slug treatment. 

As mentioned earlier that the zeta potentials of the cation/minerals are consistently and slightly more negative than that in the case of SO_4_^2−^, which is opposite to what is expected. This actually agrees with the suggested mechanism where in the first step after adsorption of SO_4_^2−^, a negative-charged compound is formed, which then is neutralized by the neighboring Al, resulting in ring formation [41].

#### 3.3.3. NaCl Brine

While flooding with NaCl brine, NPs’ breakthrough occurred at the same time and had almost the same shape as the tracer indicating minimum NP/mineral interaction (Figure 2c). The highest double layer repulsion was observed, relative to that for all the other brines (Figure 5). A declining of the average pH profile was also observed. The pH was approximately 7 in the pre-flush stage and was reduced to about 6.5 in the post-flush stage, however a slight increase in pH was observed in the slug window (Figure 6c). In contrast, other brines investigated here showed an increasing pH profile (apart from during the slug injection window (Figure 6)) to different degrees. Ca^2+^ concentration, in general, showed a slight increasing trend from an average of about 0.001 mol/L to an average of approximately 0.002 mol/L (Figure 9), which may relate to the slight reduction of the pH. 

The measured zeta potential of Na brine/minerals was more negative than that for other brines. Reid [43] and Hanna and Somasundaran [44] explained the source of the acidity. Clay minerals have exchangeable cations, so addition of NaCl follows this equation:

(7) $$H-Kaolinite+Na^{+}\to Na-Kaolinite+H^{+}.$$

The released hydrogen ions are related to the ion exchange capacity of the clay minerals and this process is a fast one. This process changes the surface charge distribution, hence affecting the adsorption of material such as for example NPs. In the case of Na brine, the lowest adsorption of NPs (~34%) was observed. The two other observations that may support the exchange mechanism are the domination of the repulsive double layer (Figure 5) and highest negative zeta potential for NPs/Berea in presence of NaCl. In Figure 6, all floods except Na flooding show similar behavior: the pH stabilizes in the pre-flush region followed by a decline after nanofluid injection. Thereafter, the pH rises and then stabilizes in the post-flush region. 

The results from this work help to identify the effect of major ions present in seawater on performance of NPs adsorption and mechanisms for NP/mineral interaction by selecting major constituents of seawater. Thus, this study improves the understanding of the influence of the individual ions on the NPs/mineral interaction and their mechanism(s). For each of the single salt brine, the salinity varied as shown in Figure 3. This study was performed at ambient conditions and may be considered as a first step of the influence of seawaters’ major ions on NP interactions with the minerals in absence of oil. So, future work may consider this approach in presence of oil. This would enable studying oil recovery along with NP adsorption and NP/mineral interactions.

## 4. Summary and Conclusions

This study investigated the effect of single salt brines, MgCl_2_, Na_2_SO_4_ and NaCl, as stabilizing fluids which are the major ion constituents of SSW on silica NP performance. The results from this study show that:

1. All the used single salt brines showed reduced mineral dissolution, however to different degrees. This is related to the degree of adsorption on the minerals, where the highest were about 82% in the cases of Mg and SO_4_ single brines, compared to about 34% in the case of Na single brine. It is demonstrated that specific ions rather than brine ionic strength/salinity are the major influencing factor on adsorption of silica NPs on Berea mineral surface.
2. The possible mechanism for the influence of Mg^2+^ on the adsorption is due to renewing and increasing of the contacted surface area at the mineral surfaces by a possible exchange reaction between Ca^2+^ and Mg^2+^. The mechanism in the case of SO_4_^2−^ ions, is due to their adsorption on kaolinite and release of OH^−^, which is followed by neutralization of the resulted negative charge by participation of neighboring Al ions resulting in ring formation, as suggested in the literature. This may also be supported by the increasing trends of the pH and the zeta potentials of the cations/minerals which are more negative than that in the case of SO_4_ brine. Another possible supporting point is that in the case of SO_4_ brine, the double layer interaction is less repulsive than those of the other brines.
3. Clay minerals have exchangeable cations so for the addition of sodium brine, an exchange between Na^+^ with H^+^ releases hydrogen ions. This process changes the surface charge distribution; hence affecting the adsorption of materials such as NPs. This is supported by that in the case of Na brine, the lowest adsorption of NPs (~34%) occurred. The two other observations that may support the exchange mechanism are the domination of the repulsive double layer (Figure 5) and that Na brine has the highest negative zeta potential, for NPs/Berea in presence of NaCl as stabilizing fluid.

The results from this study can aid in design of nanofluid flooding in sandstone reservoirs. The presence of Mg^2+^ and SO_4_^2−^ ions in the brine used for preparing nanofluids may improve the adsorption of silica NPs injected into sandstone reservoirs for controlling formation damage.

## Figures and Tables

**Table 1:** Ion concentration the brines.

Ion	SSW (mol/L)	Na Brine (mol/L)	Mg Brine (mol/L)	SO_4_ Brine (mol/L)
HCO^3−^	0.002	0	0	0
Cl^−^	0.525	0.400068	0.089033	0
SO_4_^2−^	0.0240	0	0	0.096
Mg^2+^	0.045	0	0.178066	0
Ca^2+^	0.013	0	0	0
Na^+^	0.450	0.400068	0	0.048
K^+^	0.010	0	0	0
Ionic strength	0.68	0.4	0.13	0.072

**Table 2:** Flooding experiments.

Name	Initial Saturating Fluid	Pre-flush Fluid	NP Slug	Post-Flush Fluid
BRT 1	SSW	Mg Brine	1 (g/L) NP in Mg Brine with 0.1 M LiCl tracer	Mg Brine
OBRT 2	SSW	SO_4_ Brine	1 (g/L) NP in SO_4_ Brine with 0.1 M LiCl tracer	SO_4_ Brine
BRT 3	SSW	Na Brine	1 (g/L) NP in Na Brine with 0.1 M LiCl tracer	Na Brine
BRT 4	SSW	SSW	1 (g/L) NP in SSW with 0.1 M LiCl tracer	SSW

SSW: Synthetic seawater

**Table 3:** Percent of nanoparticles retained/adsorbed in the core.

Experiment	% Nanoparticles Retained in the Core
BRT1 SSW Slug	81.7
BRT2 MG Slug	81.3
BRT3 SO_4_ Slug	68.2
BRT4 Na Slug	33.9

**Table 4:** Particle size of the dispersed silica NPs.

Dispersing Fluid	Average Hydrodynamic Radius of the NPs (nm)
SSW	28.2
Na Brine	19.6
Mg Brine	19.4
SO_4_ Brine	19.5

## References

[1] Amirian E., Dejam M., Chen Z. (2018). Performance forecasting for polymer flooding in heavy oil reservoirs. Fuel.

[2] Saboorian-Jooybari H., Dejam M., Chen Z. (2016). Heavy oil polymer flooding from laboratory core floods to pilot tests and field applications: Half-century studies. J. Pet. Sci. Eng..

[3] Mashayekhizadeh V., Kord S., Dejam M. (2014). Eor potential within Iran. Spec. Top. Rev. Porous Media Int. J..

[4] Ayatollahi S., Zerafat M.M. Nanotechnology-assisted eor techniques: New solutions to old challenges. Proceedings of the SPE International Oilfield Nanotechnology Conference and Exhibition.

[5] Al-Anssari S., Wang S., Barifcani A., Lebedev M., Iglauer S. (2017). Effect of temperature and sio 2 nanoparticle size on wettability alteration of oil-wet calcite. Fuel.

[6] Li S., Torsæter O. Experimental investigation of the influence of nanoparticles adsorption and transport on wettability alteration for oil wet berea sandstone. Proceedings of the SPE Middle East Oil & Gas Show and Conference.

[7] Abhishek R., Kumar G.S., Sapru R. (2015). Wettability alteration in carbonate reservoirs using nanofluids. Pet. Sci. Technol..

[8] Alramadan H.A. (2016). Experimental Evaluation of Surface Treated Nanoparticles and Their Effect on Wettability Alteration of Carbonate Surfaces and Oil-Brine Interfacial Tension.

[9] Olayiwola S.O., Dejam M. (2019). Mathematical modelling of surface tension of nanoparticles in electrolyte solutions. Chem. Eng. Sci..

[10] Wasan D.T., Nikolov A.D. (2003). Spreading of nanofluids on solids. Nature.

[11] Zhang H., Nikolov A., Wasan D. (2014). Enhanced oil recovery (eor) using nanoparticle dispersions: Underlying mechanism and imbibition experiments. Energy Fuels.

[12] Abhishek R., Hamouda A.A., Abdulhameed F.M. (2019). Adsorption kinetics and enhanced oil recovery by silica nanoparticles in sandstone. Pet. Sci. Technol..

[13] Yuan B., Ghanbarnezhad Moghanloo R., Pattamasingh P. Applying method of characteristics to study utilization of nanoparticles to reduce fines migration in deepwater reservoirs. Proceedings of the SPE European Formation Damage Conference and Exhibition.

[14] Arab D., Pourafshary P. (2013). Nanoparticles-assisted surface charge modification of the porous medium to treat colloidal particles migration induced by low salinity water flooding. Colloids Surf. A Physicochem. Eng. Asp..

[15] Yuan B., Moghanloo R.G., Wang W. (2018). Using nanofluids to control fines migration for oil recovery: Nanofluids co-injection or nanofluids pre-flush?—A comprehensive answer. Fuel.

[16] Abhishek R., Hamouda A.A. (2017). Effect of various silica nanofluids: Reduction of fines migrations and surface modification of berea sandstone. Appl. Sci..

[17] Abhishek R., Hamouda A.A., Ayoub A. (2018). Effect of silica nanoparticles on fluid/rock interactions during low salinity water flooding of chalk reservoirs. Appl. Sci..

[18] Abhishek R., Hamouda A.A., Murzin I. (2018). Adsorption of silica nanoparticles and its synergistic effect on fluid/rock interactions during low salinity flooding in sandstones. Colloids Surf. A Physicochem. Eng. Asp..

[19] Olayiwola S.O., Dejam M. (2019). A comprehensive review on interaction of nanoparticles with low salinity water and surfactant for enhanced oil recovery in sandstone and carbonate reservoirs. Fuel.

[20] Zahid A., Shapiro A.A., Skauge A. Experimental studies of low salinity water flooding carbonate: A new promising approach. Proceedings of the SPE EOR Conference at Oil and Gas West Asia.

[21] Morrow N., Buckley J. (2011). Improved oil recovery by low-salinity waterflooding. J. Pet. Technol..

[22] Mahani H., Keya A.L., Berg S., Bartels W.B., Nasralla R., Rossen W.R. (2015). Insights into the mechanism of wettability alteration by low-salinity flooding (lsf) in carbonates. Energy Fuels.

[23] Jackson M., Vinogradov J., Hamon G., Chamerois M. (2016). Evidence, mechanisms and improved understanding of controlled salinity waterflooding part 1: Sandstones. Fuel.

[24] Rostami P., Mehraban M.F., Sharifi M., Dejam M., Ayatollahi S. (2019). Effect of water salinity on oil/brine interfacial behaviour during low salinity waterflooding: A mechanistic study. Petroleum.

[25] Khilar K.C., Fogler H.S. (1984). The existence of a critical salt concentration for particle release. J. Colloid Interface Sci..

[26] Kia S., Fogler H., Reed M. (1987). Effect of ph on colloidally induced fines migration. J. Colloid Interface Sci..

[27] Rosenbrand E., Kjøller C., Riis J.F., Kets F., Fabricius I.L. (2015). Different effects of temperature and salinity on permeability reduction by fines migration in berea sandstone. Geothermics.

[28] Bhattacharya S., Paitaridis J., Pedler A., Badalyan A., Yang Y., Carageorgos T., Bedrikovetsky P., Warren D., Lemon N. Fines mobilisation by low-salinity water injection: 3-point-pressure tests. Proceedings of the SPE International Conference and Exhibition on Formation Damage Control.

[29] Mainwaring J. Losal: Bp’s Low-Salinity Enhanced Oil Recovery Technology. www.rigzone.com.

[30] Li S., Hadia N.J., Lau H.C., Torsæter O., Stubbs L.P., Ng Q.H. Silica nanoparticles suspension for enhanced oil recovery: Stability behavior and flow visualization. Proceedings of the SPE Europec featured at 80th EAGE Conference and Exhibition.

[31] Hendraningrat L., Li S., Torsæter O. (2013). A coreflood investigation of nanofluid enhanced oil recovery. J. Pet. Sci. Eng..

[32] Torsater O., Li S., Hendraningrat L. A coreflood investigation of nanofluid enhanced oil recovery in low-medium permeability berea sandstone. Proceedings of the SPE International Symposium on Oilfield Chemistry.

[33] Hendraningrat L., Torsæter O. (2014). Effects of the initial rock wettability on silica-based nanofluid-enhanced oil recovery processes at reservoir temperatures. Energy Fuels.

[34] Monfared A.D., Ghazanfari M., Jamialahmadi M., Helalizadeh A. (2015). Adsorption of silica nanoparticles onto calcite: Equilibrium, kinetic, thermodynamic and dlvo analysis. Chem. Eng. J..

[35] Abhishek R. (2019). Interaction of Silica Nanoparticles with Chalk and Sandstone Minerals: Adsorption, Fluid/Rock Interactions in the Absence and Presence of Hydrocarbons. Ph.D. Thesis.

[36] Dunphy Guzman K.A., Finnegan M.P., Banfield J.F. (2006). Influence of surface potential on aggregation and transport of titania nanoparticles. Environ. Sci. Technol..

[37] Zhang T., Murphy M.J., Yu H., Bagaria H.G., Yoon K.Y., Nielson B.M., Bielawski C.W., Johnston K.P., Huh C., Bryant S.L. (2015). Investigation of nanoparticle adsorption during transport in porous media. SPE J..

[38] Hamouda A.A., Valderhaug O.M. (2014). Investigating enhanced oil recovery from sandstone by low-salinity water and fluid/rock interaction. Energy Fuels.

[39] Hamouda A.A., Maevskiy E. (2014). Oil recovery mechanism (s) by low salinity brines and their interaction with chalk. Energy Fuels.

[40] Kingston F., Posner A., Quirk J. (1972). Anion adsorption by goethite and gibbsite: I. The role of the proton in determining adsorption envelopes. J. Soil Sci..

[41] Rao S.M., Sridharan A. (1984). Mechanism of sulfate adsorption by kaolinite. Clays Clay Miner..

[42] Rajan S. (1978). Sulfate adsorbed on hydrous alumina, ligands displaced, and changes in surface charge 1. Soil Sci. Soc. Am. J..

[43] Reid V., Longman G.F., Heinerth E. (1968). Tenside. Anionic detergents by two-phase titration (II). Tenside.

[44] Hanna H., Somasundaran P. (1979). Equilibration of kaolinite in aqueous inorganic and surfactant solutions. J. Colloid Interface Sci..

